# Relationship between Irisin Concentration and Serum Cytokines in Mother and Newborn

**DOI:** 10.1371/journal.pone.0165229

**Published:** 2016-11-09

**Authors:** Maria Hernandez-Trejo, Gerardo Garcia-Rivas, Alejandro Torres-Quintanilla, Estibalitz Laresgoiti-Servitje

**Affiliations:** 1 Neurobiology of Development, Instituto Nacional de Perinatologia, Mexico City, Mexico; 2 Catedra de Cardiologia, Escuela Nacional de Medicina, Tecnologico de Monterrey, Monterrey, México; 3 Centro de Investigacion Biomedica, Hospital Zambrano-Hellion, Tecnologico de Monterrey, San Pedro Garza-Garcia, Mexico; 4 Basic Medical Sciences, TEC-ABC School of Medicine, Tecnologico de Monterrey, Mexico City, Mexico; Medical Clinic, University Hospital Tuebingen, GERMANY

## Abstract

**Introduction:**

Irisin is considered to be a myokine and adipokine that may also participate in reproductive functions, as it increases significantly throughout pregnancy. However, the regulation of circulating irisin and its relationship with other cytokines has not been assessed thus far in pregnant women and their offspring.

**Objective:**

The aim of this study was to evaluate differences in irisin and cytokine concentrations between women at the end of pregnancy and their offspring, as well as the relationship between maternal and newborn irisin and maternal and newborn biomarkers.

**Methods:**

Twenty-eight mother/newborn pairs were included in this study. The following biomarkers were evaluated in maternal venous and arterial umbilical cord blood samples: irisin, 27 cytokine panel, total antioxidant capacity (TAC), total plasma protein, and free fatty acid concentration.

**Results:**

The newborns had significantly lower irisin concentrations compared to their mothers (p = 0.03), but this difference was present only in babies born from mothers without labor prior to cesarean section delivery (p = 0.01). No significant differences in maternal and newborn irisin concentrations were found between diabetic and non-diabetic mothers or between overweight/obese and normal weight mothers. A significant positive correlation was found between TAC level and irisin concentration in newborns. Maternal and newborn interleukin (IL)-1β, IL-1RA, IL-5, IL-7, and interferon gamma-induced protein (IP)-10 levels were significantly positively correlated with irisin concentrations in both study groups. In addition, maternal IL1β, IL-5, IL-7, and IP-10 levels positively predicted maternal irisin concentrations. Furthermore, arterial cord blood TAC and IL-1β and IL1-RA levels positively predicted newborn irisin concentrations. Multiple regression analyses showed that maternal IL-13 negatively predicted offspring irisin levels (p = 0.03) and that maternal IL-1β positively predicted newborn irisin concentrations (p = 0.046).

**Conclusion:**

No evidence was found that serum irisin concentrations in mothers at pregnancy termination or those of their newborns correlated with maternal body mass index, the presence of diabetes mellitus, or free fatty acid levels. However, the results of this study indicated that cytokines might predict irisin concentration in mothers and their offspring, although interactions between irisin levels during pregnancy and the newborn have not yet been fully elucidated.

## Introduction

In 2012, Boström et al. discovered irisin, a myokine capable of inducing fat browning and increasing energy expenditure [1]. Some studies refer that this myokine is secreted after exercise due to proteolytic cleavage of the membrane protein fibronectin-type III domain-containing protein 5 (FNDC5)[1–3]. Other researchers have only found an increase in irisin during acute exercise in seniors[4]. Moreover, some authors have failed to demonstrate an effect of exercise on irisin concentrations [5–7], indicating that the irisin-exercise relationship is not completely understood.

Raschke et al. showed a mutation (ATG to ATA) in the conserved start codon of the FNDC5 gene in humans that might account for a very low FNDC5 mRNA translation into protein[7]. Even though irisin appears to have a non-canonical ATA translation start in humans, the presence of circulating irisin has been confirmed by mass spectrometry [3]. Since its discovery, extensive research has been carried out to understand the role of irisin in disorders associated with insulin resistance, such as obesity and diabetes mellitus (DM) [6, 8, 9].

Pregnancy is known to be a state of progressive insulin resistance during which levels similar to those of DM patients are reached [10]. Circulating levels of irisin are also higher in pregnant women than in non-pregnant women [11]. Interestingly, irisin levels are lower in pregnant women with gestational DM (GDM) compared to body mass index (BMI)-matched glucose tolerant patients [12–14]. Maternal metabolic alterations can lead to complications such as macrosomia in newborns, and recent reports have shown that newborn irisin levels correlated with birthweight [15]. In addition, lower irisin levels have been found in cord blood samples from newborns with intrauterine growth restriction, which may result in less browning of adipose tissue in these babies [16]. Thus, irisin might play an important role in the regulation of maternal–fetal glucose homeostasis.

Pregnancy is also associated with increased oxidative stress [17, 18]. It has been reported that total antioxidant capacity (TAC) is lower during the first 30 weeks of pregnancy and increases to normal levels after reaching 37 weeks of gestation or labor [17]. Irisin has been shown to prevent oxidative stress in the liver through the inhibition of protein arginine methyltransferase-3 [19]. However, whether irisin plays a role in the redox state during pregnancy is not known.

Furthermore, normal gestation has been described as an inflammatory state [20–22]. While irisin has been labeled as a contraction-regulated myokine along with interleukin (IL)-6, IL-15, and monocyte chemotactic protein 1 [23], unlike the others, it does not function as an inflammatory cytokine and does not activate nuclear factor kappa B inflammatory pathways [24]. In fact, it might actually have anti-inflammatory properties, as it can reduce the gene expression of IL-6, tumor necrosis factor α, macrophage inflammatory protein (MIP)-1α, and MIP-1β [25]. Nonetheless, whether irisin is associated with inflammatory markers during pregnancy is unknown.

The aim of this study was to evaluate differences in irisin concentrations between women at the end of pregnancy and their newborns, as well as the relationship between maternal and offspring irisin and maternal and newborn biomarkers.

## Materials and Methods

This study was conducted at Instituto Nacional de Perinatologia, a third-level health care institution in Mexico City. The Research and Ethics Committees of the Instituto Nacional de Perinatologia "Isidro Espinosa de los Reyes" approved the study prior to patient enrollment. Written informed consent was also obtained from all the participants. Upon admission to the Labor and Delivery Unit, women who met the inclusion criteria and agreed to participate signed an informed consent form. Only patients with single, non-complicated pregnancies who had not received medication three weeks prior to delivery were enrolled in the study. Twenty-eight women were recruited, and their obstetric outcomes and clinical and demographic data were retrieved from their medical records. Maternal blood was obtained by venipuncture prior to delivery, and neonatal blood was obtained after placental delivery using a sterile method by umbilical cord arterial puncture. The blood was collected in heparinized, mineral-free, BD Vacutainer brand tubes (NJ, USA), centrifuged at 3500 RPM, and the erythrocytes and plasma were aliquoted in mineral-free vials. The samples were maintained under refrigerated conditions at all times until stored in an ultra-freezer at -86°C for further analysis. Irisin, free fatty acid, TAC, plasma protein, and cytokine concentrations of all samples were measured.

### Irisin quantitation

Irisin levels were measured using a commercial human irisin enzyme-linked immunosorbent assay (ELISA) kit (Phoenix Pharmaceuticals, Burlingame, CA, USA) according to the manufacturer’s instructions. The detection range was 0.07–1024.00 ng/mL. Each sample was assayed in duplicate and the means were used for analysis. The intra- and interassay variation coefficients were 7.0% and 9.5%, respectively.

### Cytokine quantitation

Cytokine concentrations in maternal and umbilical blood serum were measured using a 27-plex panel cytokine assay (Bio-Plex; Bio-Rad, CA, USA).

### Plasma protein concentration

Plasma proteins were quantitated using Lowry’s method [26].

### Total antioxidant capacity (TAC)

The cupric reducing antioxidant capacity spectrophotometric method was used to measure TAC, which resulted in an absorbance at 450 nm [27].

### Free fatty acid quantitation

Free fatty acid concentrations were assessed using the colorimetric method developed by Duncombe [28].

### Statistical Analysis

SPSS software version 22 (IBM, Armonk, NY, USA) was used to conduct the statistical analyses. Before executing the analyses, the assessment of univariate and multivariate outliers was performed. Data was tested for univariate and multivariate normality, linearity, and homoscedasticity. No variables required transformation. The statistical analyses performed included paired and independent samples Student’s t tests, Pearson’s correlations, and multiple linear regression analyses. A probability of α error <0.05 was considered significant

## Results

### Descriptive Statistics

Twenty-eight mother/newborn pairs were included in this study. Maternal mean age was 30.29 years (standard deviation [SD] 7.93) and mean pre-pregnancy body mass index (BMI) was 26.4 Kg/m^2^ (SD 5.2). Seventeen (60.7%) of the women enrolled in this study were obese or overweight, with a pre-pregnancy BMI ≥25 kg/m^2^; 20 participants (71.4%) were overweight at pregnancy termination. Anthropometric data of mothers and offspring are presented in Table 1. Nine patients (32.1%) had carbohydrate intolerance, GDM, or DM during the gestational period. The newborns had a mean weight of 3,010 g (SD 341.91); 14 (50%) were female and 14 (50%) were male. Irisin mean concentrations were 151.48 ng/mL (SD 134.5) in the mothers and 79.45 ng/mL (SD 14.9) in the offspring.

**Table 1:** Women and offspring anthropometric data. n = 28.

	Mean (SD)	Rank
		(min-max)
**Maternal age** (years)	30.3 (7.9)	16–43
**Pregestational weight** (Kg)	64.7 (11.7)	50–103
**Pre-pregnancy BMI** (Kg/m^2^)	26.4 (5.2)	19.5–44.5
**Weight gained during pregnancy** (Kg)	11.5 (6.2)	-4.2–25
**BMI at end of Gestation** (Kg/m^2^)	31.2 (4.5)	23.8–42.7
**Gestational age at birth** (weeks)	38.0 (1.1)	34.3–42
**Weight at birth** (grams)	2898 (341)	2355–3765
**Height at birth** (cm)	48.4 (1.9)	45–52
**Neonatal head circumference** (cm)	34.4 (1.1)	32–36
**Neonatal thoracic perimeter** (cm)	31.9 (1.5)	28–35
**Neonatal abdominal circumference** (cm)	29.8 (1.6)	25.5–33
**Neonatal ponderal Index** (grams/cm^3^x100)	2.63 (0.24)	2.13–3.02

BMI = Body Mass Index

### Inferential statistics

**Fig 1:**
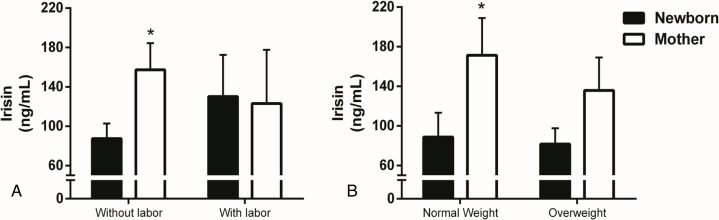
Irisin maternal levels are different from newborn when delivered without labor or when pre-pregnancy BMI is normal. Serum irisin levels of mothers and newborns according to (**A**) delivery with or without labor and to (**B**) pre-pregnancy weight status. Data are presented as mean ± SEM and * *p* < 0.05 compared to newborn using paired-samples Student’s *t*-test.

Paired samples Student’s t tests evaluated differences in irisin concentrations between maternal and umbilical cord blood samples. The newborns had significantly lower irisin concentrations compared to their mothers (94.8±77.1 vs. 151.4±127.0, ng/mL, *p*<0.03), and this difference was present only in babies born from mothers without labor prior to caesarean section as shown by paired-samples student t test after data splitting by labor (87.4±73.1 vs. 157.3±129.8, ng/mL, *p*<0.01,) (Fig 1A). The irisin levels of the neonates born with labor did not differ from the maternal concentrations (130.1±94.8 vs. 123.1±122.0, ng/mL, *p*<0.9).

It was also observed that labor increased irisin concentrations in umbilical arterial blood by nearly 40%, although this difference was not significant (87.4±73.1 vs. 130.1±94.8, ng/mL, *p*<0.2). This tendency was not observed in the maternal samples (Table 2).

**Table 2:** Differences in plasmatic irisin concentrations between maternal and umbilical cord blood according to clinical conditions and comorbidities during pregnancy (n = 28).

	Maternal sample Mean (SD)	Umbilical cord sample Mean (SD)	Mean differences	p*
	ng/ml	ng/ml		
**Total plasmatic Irisin Concentrations** (n = 28)	**151.4 (127.0)**	**94.8 (77.1)**	**56.6**	**0.03**
**Without labor before birth** (n = 24)	**157.3 (129.8)**	**87.4 (73.1)**	**69.9**	**0.01**
**With labor** (n = 4)	123.1 (122.0)	130.1 (94.8)	-7.05	0.9
p^§^	0.5	0.2		
**BMI Before pregnancy ≥ 25** (n = 17)	135.8 (137.2)	81.6 (65.5)	40.7	0.2
**< 25** (n = 11)	**171.3 (124.6)**	**88.7 (81.4)**	**82.5**	**0.04**
p^§^	0.5	0.7		
**BMI at end of pregnancy**				
**≥ 25** (n = 20)	128.4 (132.6)	85.9 (68.4)	42.4	0.1
**< 25** (n = 8)	212.0 (92.5)	118.1 (98.2)	93.8	0.057
p^§^	0.1	0.3		
**Diabetes during gestation** (n = 9)	145.1 (154.3)	83.6 (52.7)	61.5	0.2
**Without diabetes** (n = 19)	154.7 (114.8)	100.6 (88.0)	54.0	0.08
p^§^	0.8	0.5		
**Male newborn** (n = 14)	153.9 (149.5)	100.1 (71.7)	53.7	0.1
**Female newborn** (n = 14)	148.8 (103.4)	89.1 (84.8)	59.7	0.1
p^§^	0.9	0.7		
**Blood sample before caesarean section** (n = 22)	141.7 (137.6)	**79.0 (66.9)**	62.6	0.4
**Blood sample after caesarian section** (n = 6)	182.1 (87.4)	**144.3 (91.1)**	37.8	0.054
p^§^	0.4	**0.04**		

* Paired Student’s t test

§ Independent samples Student´s t test, BMI = Body mass index. Significant results (*p*≤0.05) in **bold**

As shown in Fig 1B, mothers with pre-pregnancy normal weights had significantly higher irisin concentrations compared to their offspring (171.3±124.6 vs. 88.7±81.4 ng/mL, *p*<0.04); this difference was not reflected in overweight mothers (135.8±137.2 vs. 81.6±65.5, ng/mL, *p*<0.2). There were no differences in irisin concentrations between mothers and newborns according to BMI at the end of pregnancy, GDM diagnosis, or offspring gender (Table 2).

Maternal and newborn clinical characteristics were compared between normal weight vs. overweight and diabetic vs. non-diabetic mothers. Unpaired Student’s t tests were performed to evaluate differences between those groups for the following parameters: maternal age, weight gain during pregnancy, gestational age at birth, newborn length, head circumference, thoracic perimeter, and abdominal circumference (Table 3). Diabetic mothers were nearly nine years older than non-diabetic mothers (36.2±5.2 vs. 27.6±7.6 years, *p*<0.0.5). Weight gained during pregnancy was greater when pre-pregnancy weight was normal compared to overweight (15.6±5.3 vs. 9.0 ± 5.4 kg, *p*<0.003) and in non-diabetic vs. diabetic mothers (13.8±5.5 vs. 7.2±5.1 kg, *p*<0.004). Other clinical parameters did not differ between the groups studied (Table 3).

**Table 3:** Differences in maternal and newborn characteristics between overweight and normal weight, and diabetic and non-diabetic mothers (n = 28).

	BMI Before pregnancy ≥25 (n = 17)	BMI Before pregnancy <25 (n = 11)	*p*	Diabetic Pregnancy (n = 9)	Non Diabetic Pregnancy (n = 19)	*p*	BMI ≤25at end of pregnancy (n = 20)	BMI >25 at end of pregnancy (n = 8)	*p*
**Maternal age** (years)	31.9	28.3	0.2	**36.2**	**27.6**	**0.05**	30.7	30.1	0.8
	(6.8)	(9.4)		**(5.2)**	**(7.6)**		(7.4)	(9.6)	
**Weight gained during pregnancy** (Kg)	**9.0**	**15.6**	**0.003**	**7.2**	**13.8**	**0.004**	12.2	9.8	0.3
	**(5.4)**	**(5.3)**		**(5.1)**	**(5.5)**		(6.7)	(4.6)	
**Gestational age at birth** (weeks)	37.6	38.3	0.2	37.6	38.2	0.1	38.0	37.6	0.7
	(1.2)	(0.7)		(1.0)	(1.1)		(1.2)	(0.7)	
**Weight at birth** (grams)	3020	2960	0.6	3148	2919	0.08	3028	2917	0.4
	(373)	(294)		(348)	(297)		(325)	(392)	
**Height at birth** (cm)	48.6	48.1	0.5	48.7	48.3	0.6	48.6	48.0	0.1
	(1.7)	(2.2)		(1.9)	(1.9)		(1.8)	(2.1)	
**Head circumference** (cm)	34.5	34.3	0.6	34.9	34.2	0.1	34.6	33.8	0.058
	(1.1)	(1.2)		(0.8)	(1.2)		(1.0)	(1.3)	
**Thoracic perimeter** (cm)	31.8	32.1	0.6	32.5	31.6	0.1	32.0	31.8	0.7
	(1.7)	(1.2)		(1.6)	(1.4)		(1.5)	(1.6)	
**Abdominal circumference** (cm)	29.7	30.0	0.6	30.2	29.7	0.4	29.9	29.7	0.7
	(1.6)	(1.6)		(1.4)	(1.7)		(1.7)	(1.4)	

Mean (SD) Independent samples Student’s t test. Significant results (*p*≤0.05) **in bold**.

TAC was almost 50% higher in maternal samples than in cord blood samples (1.339±0.152 vs. 0.7067±0.428 μmol trolox equivalents/mL, *p*<0.000). These differences were also present when analyzing TAC by pre-pregnancy and end of pregnancy BMI (data not shown). Mean protein concentration was 64.77±6.54 mg/mL in the maternal samples and 26.1±13.3 mg/mL in the newborn samples. Cord blood sample protein concentration was almost 60% lower compared to that of the mother, which might explain the differences in TAC values.

To study whether irisin levels were dependent on other clinical variables or biomarkers, multiple linear regression and Pearson’s correlation were used. Pearson’s correlation revealed a significant positive correlation between offspring TAC and irisin concentrations. On the other hand, maternal and newborn IL-1β, IL-1Ra, IL-5, IL-7, and interferon gamma-induced protein 10 had significant positive correlations with irisin concentrations in both study groups. Cord blood IL-6, interferon γ, monocyte chemotactic and activating factor (MCAF), and Regulated on Activation Normal T-cell expressed and secreted cytokine (RANTES) had significant positive correlations with offspring irisin concentrations, whereas maternal IL-10 correlated positively with maternal irisin concentrations. Conversely, free fatty acid concentrations did not correlate with irisin concentrations in mothers (r = 0.02, p = 0.8) or newborns (r = 0.34, p = 0.06) (data not shown).

Multiple lineal regression analyses were performed to evaluate whether TAC or cytokines predict maternal or offspring irisin concentrations. Four independent variables were entered per analysis. The results indicated that maternal IL-1β, IL-5, IL-7, and IP-10 positively predicted maternal irisin concentrations and that arterial cord blood TAC, IL-1β, and IL1-RA, positively predicted newborn irisin concentrations. On the other hand, newborn platelet-derived growth factor, IL-7, and MCAF negatively predicted newborn irisin concentrations. While cord blood TAC positively predicted newborn irisin concentrations, TAC did not predict maternal irisin levels. Table 4 shows Pearson’s *r* values, B coefficients, and significant values for regression analyses between maternal biomarkers and maternal irisin and between newborn biomarkers and newborn irisin concentrations. Notably, total plasma protein levels did not significantly predict or correlate with irisin concentrations in either the mothers or the offspring.

**Table 4:** Multiple Regressions and Pearson Correlations between maternal and newborn Irisin (ng/mL) and maternal/offspring conditions (n = 28).

	MULTIPLE LINEAL REGRESSIONS		PEARSON CORRELATIONS	
	B/p*		r/p*	
	Maternal Plasma Irisin	Offspring Plasma Irisin	Maternal Plasma Irisin	Offspring Plasma Irisin
**CLINICAL CHARACTERISTICS**				
**Maternal age** (years)	-0.9 / 0.8	0.1 / 0.9	-0.02 / 0.9	0.09 / 0.6
**BMI Before pregnancy** (Kg/m^2^)	4.6 / 0.7	-14.2 / 0.09	0.1 / 0.5	-0.07 / 0.3
**Weight gain during pregnancy** (Kg)	0.4 / 0.9	-5.4 / 0.1	-0.07 / 0.7	-0.03 / 0.8
**BMI at end of pregnancy** (Kg/m^2^)	-1.4 / 0.9	12.1 / 0.1	0.08 / 0.6	-0.04 / 0.8
**Gestational age at birth** (weeks)	-22.7 / 0.3	-6.4 / 0.6	-0.1 / 0.4	0.03 / 0.8
**Weight at birth** (grams)	-1 / 0.4	0.03 / 0.6	-.008 / 0.6	0.07 / 0.7
**Height at birth** (cm)	-10.7 / 0.5	13.0 / 0.2	-0.1 / 0.5	0.02 / 0.2
**Head circumference** (cm)	-35.1 / 0.3	-42.7 / 0.1	-0.2 / 0.2	-0.16 / 0.3
**Thoracic perimeter** (cm)	-28.6 / 0.2	1.1 / 0.9	-0.2 / 0.2	0.02 /0.8
**Abdominal circumference** (cm)	33.9 / 0.1	14.7 / 0.2	0.05 / 0.7	0.2 / 0.2
**Significant PLASMA OXIDATIVE BIOMARKERS**				
**TAC** (μmol of trolox/mL)	-30.3 / 0.6	**120.0 / 0.005**	-0.1 / 0.3	**0.6 / 0.000**
**PLASMA CYTOKINES & CHEMOKINES (ng/mL)**				
**PDGF**	-0.02 / 0.4	**-0.03 / 0.05**	0.3 / .1	0.2 / 1
**IL-1B**	**100.9 / 0.03**	**120.3 / 0.04**	**0.53 / 0.003**	**0.42 / 0.02**
**IL-1RA**	0.4 / 0.8	**0.5 / 0.01**	**0.46 / 0.01**	**0.45 / 0.01**
**IL-4**	-4.2 / 0.2	-6.3 / 0.5	0.32 / 0.8	0.1 / 0.3
**IL-5**	**32.2 / 0.008**	19.1 / 0.1	**0.45 / 0.01**	**0.47 / 0.09**
**IL-6**	1.7 / 0.2	7.9 / 0.2	0.15 / 0.4	**0.46 / 0.01**
**IL-7**	**22.3 / 0.04**	**-1.1 / 0.04**	**0.52 / 0.03**	**-0.41 / 0.02**
**IL-10**	2.7 / 0.9	40.2 / 0.07	**0.41 / 0.02**	0.29 / 0.1
**IL-13**	4.3 / 0.4	8.9 / 0.1	0.2 / 0.2	-0.04 / 0.8
**GCSF**	1.4 / 0.2	-0.68 / 0.1	0.22 / 0.2	-0.25 / 0.1
**IFN γ**	0.03 / 0.9	0.5 / 0.06	0.34 / 0.06	**0.43 / 0.01**
**IP-10**	**0.07 / 0.01**	0.03 / 0.1	**0.46 / 0.01**	**0.39 / 0.03**
**MCAF**	0.4 / 0.8	**-1.6 / 0.005**	0.29 / 0.1	**-0.48 / 0.008**
**MIP-1B**	2.0 / 0.2	1.3 / 0.1	0.30 / 0.1	0.03 / 0.8
**RANTES**	-0.08 / 0.1	-0.009 / 0.08	0.10 / 0.6	**-0.37 / 0.04**

* Significant results (*p*≤0.05) in **bold**.

Lastly, multiple regression analyses were carried out to assess whether maternal cytokine concentrations predict newborn irisin concentrations. Significant results were found for IL-13, which negatively predicted offspring irisin levels (B = -11.79, *p* = 0.03), and maternal IL-1β, which positively predicted offspring irisin concentrations (B = 160.74, *p* = 0.046).

## Discussion

In accordance with previous reports, we found that irisin concentrations were lower in newborns compared with their mothers [29, 30]; however, this difference was not present in mother–newborn pairs who did not go through labor (Fig 1A). In this regard, has been established that irisin secretion is increased by exercise [1, 3]; therefore, the physical stress related to labor [31], possibly mimicking physical activity, might explain the higher irisin levels in those newborns.

Several groups have characterized irisin changes in pathological conditions such as obesity and DM. It has been reported that serum irisin concentrations in non-pregnant obese patients are elevated and positively correlated with BMI [32, 33]. In type 2 DM studies, studies have reported that non-pregnant patients were found to have lower irisin levels, showing a potential role in insulin resistance [6, 34, 35]. However, Al-Daghri et al. found significantly higher levels of irisin in non-pregnant women with type II diabetes mellitus compared to controls and it was not associated to the level of habitual physical activity in these patients [36]. Alterations in circulating irisin during pregnancy are less clear. While two different groups found no correlation between BMI and irisin in obese pregnant patients [11, 37], lower levels of irisin were reported in GDM patients by two other studies [12, 30]. Another study found no difference in circulating irisin between healthy and GDM pregnancies; however, irisin was independently predicted by fasting glucose [38]. Similarly, Garces et al. found a positive linear correlation between irisin and the homeostatic model assessment index for insulin resistance [11].

The results of the current study showed higher irisin levels in mothers compared to newborns in patients with healthy pre-pregnancy weights compared to overweight patients. In accordance with previous reports [11, 37], there were no differences in maternal irisin concentrations related to BMI (Table 1). Our results are similar to those of Ebert et al. [38], as we found no differences in irisin concentrations between pregnant women with and without GDM or in their offspring. We also found no correlation between free fatty acids and irisin levels in mothers and offspring. Likewise, Lopez-Legarrea et al. found no correlation between irisin levels and free fatty acids [8], and Wang et al. provided evidence that a relatively higher concentration of irisin had no effect on lipolysis in mature 3T3-L1 adipocytes [39].

The newborn irisin concentrations in our study were similar to those reported by Baka et al. [15]. However, irisin levels did not correlate with newborn weight in this study, which differed from the results of Çağlar et al. and Baka et al. [15, 29]. We also found no correlation between umbilical artery irisin and gestational age, which is similar to the results of Ebert el al. [29, 38]. Under normal conditions, arterial cord blood protein concentration may be around 28–53% lower than the concentration in maternal serum [40], which is similar to what was found in the current study.

Several authors believe that newborn irisin may be maternally inherited [15, 41]. According to Al Gadhri, irisin concentrations in the newborn are promoted by maternal factors that participate in energy regulation, specifically, high-density lipoprotein cholesterol [41]. This biomarker was not evaluated in this study. Nonetheless, we found that newborn TAC and IL-1β predicted and positively correlated with newborn irisin levels. While newborn IL-1β, MCAF, and IL-7 negatively correlated with newborn irisin concentrations in our study, Al-Daghri et al. found that newborn angiotensin II predicts irisin levels in the offspring [41].

We found that lower levels of maternal IL-13 are associated with higher offspring irisin concentrations. To our knowledge, there are no published studies on this subject, and we have no clear explanation for it. IL-13 has IL-4-like activities and has been related to atopic disease [42]. It is considered a Th2-type cytokine and, similar to IL-10, it may inhibit macrophage activity [43]. This cytokine is produced in considerable amounts by the placenta during pregnancy, and lower IL-13 levels in the newborn have been related to increased risk of atopy [42].

IL-13 is also considered to be a myokine, and it might promote increased glucose uptake and glucose oxidation [44, 45]. Contrary to meteorin-like myokine, a factor secreted in adipose tissues and muscles after cold exposure exercise, irisin does not increase gene expression of IL-13 or lead to alternative macrophage activation [46]. In the current study, maternal IL-13 inversely correlated with offspring irisin. Although irisin has not been associated with IL-1β in previous studies, we found that maternal IL-1β positively predicted offspring irisin concentrations. Irisin has mostly been associated with IL-6, IL-8, and IL-15 because contractile activity might induce expression of these cytokines in skeletal muscle [47]; however, there is little information regarding the relationship of irisin with other cytokines. Clearly, the relationship between maternal cytokine concentrations and newborn irisin concentrations requires further investigation.

This study has some limitations. First, due to the rigorous inclusion criteria, few patients met the criteria for enrollment and, although we had significant results, we worked with a small sample size. Certainly, a larger sample size could have increased the possibility of achieving significant results, especially in interactions that produced small effects.

It is noteworthy that contradictory findings have been reported regarding the nature and concentration of serum FNDC5/Irisin [48] and several articles have not only questioned the relevance of irisin in humans but the degree of shedding of soluble irisin as well [5]. Besides, research in humans using recombinant irisin does not reflect the same functions that have been reported in mice, especially regarding the brightening of adipocytes [7]. Some studies have also raised doubt concerning irisin secretion by skeletal muscle cells [23] and others refer that this myokine may be present in such low concentrations that may render a physiological role for irisin very unlikely [49]. Additionally, the validity of the commercially available irisin quantitation ELISA methods has been questioned, arguing that they may be reporting cross-reacting antibodies and a potential null mutation on human FNDC5 [49, 50]. Because of the possible deficiencies in irisin detection methods reported in previous studies [49], in this work we used the irisin detection method whose antibody has been validated by three different groups by immunohistochemistry [51–53], the commercial ELISA kit by Phoenix Pharmaceuticals [54].

In spite of studies challenging the possible effects of irisin, several others support its physiological roles. Recently, a paper using mass spectroscopy demonstrated that human irisin is translated from a non-canonical start codon, it circulates in plasma and is regulated by aerobic exercise [3]. Moreover, studies have shown that adipose tissue expresses and secretes irisin. Interestingly, it may be increased in patients with metabolic disturbances, such as obesity; and weight loss and fat mass reduction can be responsible for a decrease in FNDC5/irisin concentration [48]. However, we did not find a correlation between irisin and BMI or the presence of DM in this study. It is evident that further studies are warranted in order to clarify the controversy that remains in the literature regarding FNDC5/irisin.

## Conclusions

A growing body of evidence has revealed an important role for irisin in metabolism. However, in the present study, we found no proof that serum irisin concentrations in mothers at pregnancy termination or in their newborns correlated with BMI, the presence of DM, levels of free fatty acids, or the offspring’s gestational age or birth weight. Nonetheless, interesting findings came about regarding the relationships between irisin and other biomarkers, such as TAC and cytokines, the latter being relevant for fetal–placental development.

As irisin has recently been considered to be an adipo-myokine and it might be closely related to some cytokines, it might be a participant in endocrine and immune system cross-talk during pregnancy. Unfortunately, the mechanisms originating irisin production and its interactions with other biomarkers during the gestational period and in the newborn still remain unascertained.
